# Advances in Understanding and Harnessing the Molecular Regulatory Mechanisms of Vegetable Quality

**DOI:** 10.3389/fpls.2022.836515

**Published:** 2022-03-08

**Authors:** Luyao Gao, Ning Hao, Tao Wu, Jiajian Cao

**Affiliations:** ^1^College of Horticulture, Hunan Agricultural University, Changsha, China; ^2^Engineering Research Center for Horticultural Crop Germplasm Creation and New Variety Breeding, Ministry of Education, Changsha, China; ^3^Key Laboratory for Vegetable Biology of Hunan Province, Changsha, China; ^4^College of Horticulture and Landscape Architecture, Northeast Agricultural University, Harbin, China; ^5^Graduate School of Agricultural and Life Sciences, The University of Tokyo, Tokyo, Japan

**Keywords:** gene manipulation, vegetables, quality, *Brassicaceae*, *Solanaceae*, *Cucurbitaceae*

## Abstract

The quality of vegetables is facing new demands in terms of diversity and nutritional health. Given the improvements in living standards and the quality of consumed products, consumers are looking for vegetable products that maintain their nutrition, taste, and visual qualities. These requirements are directing scientists to focus on vegetable quality in breeding research. Thus, in recent years, research on vegetable quality has been widely carried out, and many applications have been developed via gene manipulation. In general, vegetable quality traits can be divided into three parts. First, commodity quality, which is most related to the commerciality of plants, refers to the appearance of the product. The second is flavor quality, which usually represents the texture and flavor of vegetables. Third, nutritional quality mainly refers to the contents of nutrients and health ingredients such as soluble solids (sugar), vitamin C, and minerals needed by humans. With biotechnological development, researchers can use gene manipulation technologies, such as molecular markers, transgenes and gene editing to improve the quality of vegetables. This review attempts to summarize recent studies on major vegetable crops species, with *Brassicaceae, Solanaceae*, and *Cucurbitaceae* as examples, to analyze the present situation of vegetable quality with the development of modern agriculture.

## Introduction

With the improvement of living standards, an increasing number of people are paying close attention to the nutritional components and taste of vegetables. On the market, high-quality vegetables, such as broccoli, which contains high glucoraphanin levels, and cucumbers, which have glossy peels, are popular with consumers (Jin et al., 2014; Zhang et al., 2021). With the development of society and the improvement of people’s living standards, consumers not only seek vegetables to eat but also have a wide range of choices of vegetables, with the accompanying expectations that they are attractive, nutritionally rich, delicious, fresh, safe and environmentally friendly. Given the requirements for the quality of vegetables, researchers have focused their efforts on systematic studies about the nutritional value of vegetables. In general, vegetable quality includes sensory characteristics and biochemical properties, which can be divided into three aspects, i.e., appearance, texture, and flavor, whereas biochemical properties can be used in the analysis and evaluation of the nutritional value and safety of vegetable crop species (Aung and Chang, 2014; Liu et al., 2017; Bort et al., 2019; Zhao et al., 2019).

**FIGURE 1:**
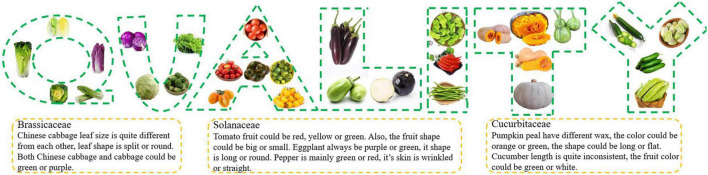
Overview of the different vegetable commodity characteristics, with *Brassicaceae*, *Solanaceae*, and *Cucurbitaceae* as examples. Extensive research has been performed to optimize leaf size (e.g., split or round Chinese cabbages), leaf color (e.g., green or purple cabbages), fruit color (e.g., red, yellow or green tomatoes, purple or green eggplants, and orange or green pumpkins), fruit shape (e.g., big or small tomatoes, wrinkled or straight eggplants, and long or flat pumpkins), and fruit size (e.g., cucumbers of different lengths). Vegetables are usually optimized to meet consumer expectations for product quality, including appearance, taste, and texture.

The quality evaluation criteria of different vegetables and the regulatory mechanisms behind these traits are quite different (Figure 1). Therefore, summarizing the quality characteristics of different crop species is necessary to improve vegetable quality by gene manipulation. Currently, in addition to traditional radiation mutagenesis, chemical mutagenesis, and hybrid breeding methods, genetic manipulation is gradually being applied in the innovation of vegetable crop germplasm resources, but its specific application in quality improvement remains unclear. This paper summarizes the application of genetic manipulation to improve vegetable quality.

## Quality Characteristics of *Brassicaceae*

Consumers often judge the quality of vegetable crops by their appearance. For example, consumers prefer densely colored cabbage leaves because they believe that the short light time and/or short growth cycle cause(s) the color of cabbage to be light, which leads to a relatively low nutritional quality (Amagai et al., 2021). Moreover, different consumers make various choices regarding vegetable quality due to their different needs or preferences. For instance, someone may prefer loose-type cabbage because they find its leaves to be crisp and sweet when chewing. Conversely, firm cabbage has a higher plant fiber content and is softer and more flexible. Therefore, germplasm resources of different qualities must be created to align with various consumer expectations.

### Commodity Quality of *Brassicaceae*

Commodity quality refers to morphological characteristics that can be evaluated by appearance, including plant type, plant height, leaf size, leaf color, petiole characteristics, handle color, and handle shape (Cox et al., 2003; Cheng et al., 2009; Olfati et al., 2010; Kim et al., 2020). There are regional differences in the requirements for the quality of vegetables due to different eating and consumption habits. Many kinds of large-leaf Chinese cabbage can be found in northeast China, while small-leaf Chinese cabbage is most popular in southern China. For *Brassicaceae*, leaves are the most important organ because they are often edible or are involved in nutrient accumulation, and leaf size determines product yields. Current evidence shows that the *Brassicaceae* leaf area is connected with phytohormones. The transcription factor *AINTEGUMENTA* (*ANT*), which encodes the *APETALA2/ETHYLENE RESPONSE FACTOR* (*AP2/ERF*) family, responds to auxin and regulates downstream gene expression related to organogenesis and cell proliferation, indicating that *ANT* promotes leaf growth by regulating cell division (Klucher et al., 1996; Note-Wilson et al., 2010; Ding et al., 2018; Karamat et al., 2021). Three *ANT* and six *ANT-like* (*BrAIL*) genes were identified in Chinese cabbage; the expression of the *BrANT* gene and three *BrAIL* genes increased with auxin treatment, and the leaf size increased (Ding et al., 2018). *ETHYLENE RESPONSE FACTORS* (*ERFs*) encode AP2/ERF superfamily transcription factors, which are the central components of the ethylene signaling pathway (Muller and Munne-Bosch, 2015). Overexpression of *BrERF4* in *Arabidopsis thaliana* can reduce leaf size by inhibiting cell expansion. For *BrERF4* overexpression, two *EXPANSIN* (*EXP*) genes, *AtEXPA5* and *AtEXPA10*, are downregulated in *A. thaliana* (Park et al., 2012). Thus, ethylene can effectively regulate *Brassicaceae* leaf size. In addition to leaf size, leaf shape can affect consumer choice; for example, some consumers prefer split-leaf Chinese cabbage, but others prefer round leaves. *LOST MERISTEM2* (*BrLOM2*) may be the main factor regulating the phenotype of split leaves. A comparison of the round-leaf Chinese cabbage with split-leaf near isogenic lines shows that the expression of *BrLOM2* in split leaves increases synchronously with the number of leaf edge cracks (Shu-juan et al., 2016). Moreover, the plant surface is crucial in determining vegetable quality, including brightness and color. The wax content in *Brassicaceae* is the main factor controlling brightness, and it also affects stress defense. One principal component analysis of different waxy materials showed that the expression of the *LIPID TRANSFER PROTEINS* (*LTP2*) gene is higher in the least waxy lines and that of the *ECERIFERUM3* (*CER3*) gene is more highly expressed in the most waxy lines in *Brassica oleracea* var. *capitata*, indicating that *LTP2* and *CER3* may be related to the wax content (Laila et al., 2017). Scanning electron microscopy and gas chromatography–mass spectrometry (GC–MS) revealed that the wax content of the *CRISPR-BoCER1* plant “*CW1-3*” is significantly lower than that of the wild type, proving that *BoCER1* is crucial in the biosynthesis of cabbage epidermal wax (Cao et al., 2021). In addition to affecting glossiness, wax-related genes play an important role in defense against adverse stresses and are worthy of further exploration. In terms of color, for example, purple leaf *Brassicaceae* varieties are popular on the market. Given the different colors of vegetables, the contents of pigments also varies. Li et al. (2019) found that the relative expression of *BASIC HELIX-LOOP-HELIX49* (*bHLH4*9) in stalks and young leaves of Zicaitai “Xianghongtai 01” is significantly higher than that in Caitai “Yinong50D,” indicating that *bHLH49* affects the color formation of Zicaitai. These studies on the appearance of *Brassicaceae* will help enrich commodity quality and provide consumers with more choices.

### Flavor Quality of *Brassicaceae*

*Brassicaceae* flavor quality includes sweetness, crispness, softness, juiciness and the lack of undesirable smells. In particular, the leaves and petioles tend to be soft, the odor should be fresh and fragrant, and the material should be tender and strong (Wright et al., 2006). Moreover, high oxygen atmospheric packaging (> 70 kPa, HOAP) can maintain vegetable quality. HOAP affects the production of hydrogen peroxide (H_2_O_2_), increases cabbage tissue firmness, and significantly reduces the contents of hemicellulose, cellulose, and lignin in the stem. Therefore, the decrease in the H_2_O_2_ signal in hemicellulose, cellulose, and lignin biosynthesis may be related to the differential accumulation of oxidative stress-related proteins that are induced by HOAP treatment (Wang et al., 2020). At present, studies about *Brassicaceae* flavor quality are limited, and the mechanism by which special flavors are formed has not yet been found.

### Nutritional Quality of *Brassicaceae*

Nutritional quality refers to nutritional value, which is mainly determined by the contents of nutrients but is also affected by the contents of harmful components and pollutant residues. The nutritional quality of *Brassicaceae* requires high contents of flavonoids, L (+)-ascorbic acid (AsA), dry matter, soluble solids, and vitamins; a moderate content of crude fiber; a low content of nitrate; and absence of pollutant residues such as pesticides. The unique nutritional quality of *Brassicaceae* is attributed to flavonoids. Watercress is a special plant rich in flavonoids. Four important watercress varieties were compared with non-heading Chinese cabbage by ultrahigh-performance liquid chromatography-electrospray ionization-tandem mass spectrometry (UHPLC–ESI–MS/MS). A total of one hundred thirty-two flavonoid metabolites were detected: eight anthocyanins, two dihydroflavonoids, three dihydroflavonols, one flavonoid, twenty-two flavonoids, eleven flavonol carbon glycosides, eighty-two flavonols and three isoflavones. Marked differences in flavonoid metabolites were found in different samples, and all of them exhibited their own unique metabolites (Ma et al., 2021). The most important nutritional quality of *Brassicaceae* is the content of AsA, which can be regenerated from monodehydroascorbate and dehydroascorbate by *MONODEHYDROASCORBATE REDUCTASE* (*MDHAR*). Overexpression of *MDHAR1* from non-heading Chinese cabbage reduces the AsA level and growth in transgenic tobacco (Ren et al., 2015). Additionally, there is a very significant negative correlation between the nitrate content and AsA content in leaf lettuce (Koyama et al., 2012). These results provide a reference for enhancing the nutritional quality of *Brassicaceae* crop species.

## Quality Characteristics of *Solanaceae*

Given that people eat only the fruits of solanaceous crop species, the key to improving the quality of solanaceous crops is to focus on fruit quality. Consumers have different evaluation standards for the fruit quality of *Solanaceae*. For example, tomatoes with uniform color, thin skin, and no cracks are more popular than their counterparts. When the taste is sweet and sour, the quality of tomatoes is considered high. Similarly, uniform color and strong luster are important qualities for peppers. Plump and juicy peppers taste better and are more popular with consumers than their counterparts. Less spicy peppers with moderate aromas sell well in northern China. However, peppers with high levels of spiciness and strong scents are highly popular among consumers in southern China, such as Sichuan and Hunan Provinces. In addition, factors such as cooking techniques and geographical restrictions lead to different consumer needs, so many varieties of solanaceous crops exist on the market.

### Commodity Quality of *Solanaceae*

Tomatoes, peppers, and eggplants are common *Solanaceae* vegetables and are important parts of our table dishes. Given that the main edible part of solanaceous crops is the fruit, fruit shape and color are important commodity qualities. For example, the best-selling tomato requires uniform coloring, bright color, small cracks in the apical pedicel, and no longitudinal or ring cracks (Yildiz and Baysal, 2007; Liu et al., 2020; Xue et al., 2020). Currently, most consumers prefer bright red or pink fruit colors, and a few prefer an orange fruit color, which varies with the changes in eating habits in different regions (Veerappan et al., 2016; Yang et al., 2019). Similar to *Brassicaceae*, pigments, such as chlorophyll, lutein, carotene, and anthocyanin, affect the color of tomatoes. The expression of the structural flavonoid genes is closely followed by *MYB12*, suggesting that *MYB12* regulates the production of flavonoids in tomato fruit by activating the transcription of the genes encoding these pathway enzymes, and *MYB12* is also reported to be suppressed in pink line tomatoes (Adato et al., 2009; Ballester et al., 2010). *SlMYB12* mutation leads to premature termination of the amino acid sequence and structural changes, resulting in a colorless epidermis phenotype in tomato fruits (Veerappan et al., 2016). Compared with yellow tomatoes, *ISOPENTENYL DIPHOSPHATE ISOMERASE 1* (*IDI1*) is a cytoplasmic enzyme involved in the biosynthesis of isoprenoids, including cholesterols, that inserts a single T base into exon 6 in apricot tomatoes, resulting in a decrease in carotenoid content and yellow pericarp (Nakamura et al., 2015; Shin et al., 2019; Chattopadhyay et al., 2021). Several genes and their mutant alleles related to carotenoid biosynthesis have been identified and characterized in tomato fruit. *PHYTOENE SYNTHASE* (*PSY1*), *CAROTENOID ISOMERASE* (*CrtISO*), *LYCOPENE BETA CYCLASE* (*CYC-B*), and *LYCOPENE E -CYCLASE* (*LCY-E*) mutations can affect the carotene content and change the color of the tomato epidermis, but the mechanisms of the formation of different colors remain unclear (Hwang et al., 2016; Yoo et al., 2017; Chen et al., 2019). For purple eggplant, pericarp color intensity is an important economic characteristic. Many factors are considered in assigning the final color intensity, one of which is the accumulation of anthocyanins and chlorophyll. Comparing two advanced purple eggplant lines, EP26 and EP28, with different pericarp color intensities, a higher anthocyanin content and lower chlorophyll content were observed in EP26, and deeper pericarp color intensity was observed at two developmental stages. In addition, comparative transcriptome analysis of EP26 and EP28 showed that 131 transcription factors, including those of the MYB, bHLH, WRKY and NO APICAL MERISTEM, *Arabidopsis* TRANSCRIPTION ACTIVATION FACTOR1/2, and CUP-SHAPED COTYLEDON (NAC) families, displayed dynamic changes, which may have been due to the changes in fruit pigment accumulation between EP26 and EP28 (Zhou et al., 2021). Although some genes related to *Solanaceae* fruit color have been clarified, the mechanism by which different colors are formed has yet to be elucidated. Further research on the molecular regulatory mechanism of color is necessary to create multicolor germplasm resources.

Tomato cracking generally occurs during fruit ripening and is affected by genetic or environmental factors such as fruit firmness, cuticle characteristics, abscisic acid (ABA), gibberellins (GAs), water, light and nutrients (Cox et al., 2003; Bargel and Neinhuis, 2005; Khadivi-Khub, 2015; Yang et al., 2016; Jiang et al., 2019). Cracking greatly reduces the edible value of tomatoes, and tomatoes with no cracks are highly popular on the market. One crack-resistance gene, *Cr3a*, was found by mapping. The water content of cracked tomato fruit during the green ripening period was significantly higher than that of the *Cr3a* crack-resistant tomato. However, no significant difference in the thickness of the cuticle or the number of epidermal cells was found between these two kinds of tomatoes, and the mechanism remains unclear (Yu et al., 2020). In addition to these genetic or environmental factors, tomato cracking is related to the pectin content. POLYGALACTURONASE (PG) can degrade the pectin backbone, and EXPANSIN (EXP) is a non-enzymatic cell wall active protein. PG and EXP cooperatively disassemble wall polysaccharide networks and contribute to the softening of fruit. By suppressing *SlPG* and *SlEXP1* expression in tomato fruit (*pg/exp*), the content of water-soluble pectin decreases in the pericarp, whereas the content of propectin increases. Although the cell wall and wax layer become thicker and the *pg/exp* fruit hardens, the rate of fruit cracking is reduced due to the firm protopectin (Jiang et al., 2019).

The texture of the fruit, especially the firmness, is the main quality of fresh tomatoes evaluated by consumers (Hongsoongnern and Chambers, 2008; Brashlyanova et al., 2014). Most consumers prefer hard tomatoes, which have better cooking characteristics. The factors affecting the firmness of tomatoes are epidermal toughness, pulp firmness and the internal structure of fruits (Chapman et al., 2012; Romero and Rose, 2019). *FIRM SKIN 1* (*FIS1*) encodes a GA2-oxidase, and changes in the level of GAs can induce parthenocarpic development and affect fruit maturity in tomatoes (Martinez-Bello et al., 2015; Li et al., 2019). Early termination of the *FIS1* gene increases the biological activity of GA, the biosynthesis of thorny and waxy layers, and the shelf life and firmness of fruits in tomato (Li et al., 2020). These changes improve the firmness of tomato fruits, thereby increasing transportability and yielding more economic benefits.

### Flavor Quality of *Solanaceae*

For tomato, the main factors affecting flavor quality are the contents of sugar and acid and the ratio of sugar to acid (Beckles, 2012). High sugar and low acid contents make the tomato taste light, whereas a low ratio of sugar to acid makes the tomato taste sour. When both are low, the fruit is tasteless. For good flavor, the fruit must have a high sugar content and high sugar:acid ratio. In recent years, given the obvious diversification of consumer groups, tomato fruits with increased acidity have been favored by special groups such as beverage lovers under the premise of a high sugar content. The cell wall *CONVERTING ENZYME INHIBITOR 1* (*SlCIF1*) gene, which is involved in tomato glucose metabolism, activates the small *HEAT SHOCK PROTEIN 17.7* (*SlHSP17.7*) gene to control the flavor of tomato. In *SlHSP17.7*-RNA interference (RNAi) lines, the sweetness of tomato is significantly decreased by modulation of the contents of sucrose and fructose (Zhang et al., 2018).

Spicy taste is a unique flavor quality of pepper. Whole-genome sequencing and assembly of the hot pepper (Mexican landrace of *Capsicum annuum* cv. CM334) revealed fifty-four *CAPSAICINOID BIOSYNTHETIC GENES* (*CBGs*) related to the spicy taste of pepper; *CBGs* are specifically expressed in the placenta of pepper, but the mechanisms behind these genes are unclear (Kim et al., 2014). *MYB31* is a transcription factor specifically expressed in pepper placenta. Transcription level analysis revealed that *MYB31* is highly coexpressed with *CBG*. Further experiments revealed that *MYB31* directly regulates *ACYLTRANSFERASE* 3(*AT3*), which exhibits developmentally regulated placenta-specific expression and participates in capsaicin biosynthesis by binding to MYB *cis*-elements. Moreover, *MYB31* directly regulates *CBG* expression and participates in capsaicin biosynthesis (Zhu et al., 2019). Consumer preferences for pepper spiciness are affected by subjective factors, so breeders can select corresponding varieties depending on the preferences of local consumers.

### Nutritional Quality of *Solanaceae*

The content of AsA is an important index of quality. *MYO-INOSITOL OXYGENASE* (*MIOX*) is a critical enzyme in the plant AsA biosynthesis pathway. *MIOX4* overexpression tomato lines show a significant increase in total ascorbate in leaves and red fruits compared to the control (Munir et al., 2020). After the knockout of *ASCORBATE OXIDASE* (*AO*) and mitochondrial *ASCORBATE PEROXIDASE* (*mitAPX*), the decreased AO enzyme activity and significantly improved AsA content in tomato fruit were observed to be correlated with *AO* gene suppression, and the downregulated *mitAPX* expression and APX enzyme activity led to an increase in the content of AsA in tomato fruits compared with the wild type, indicating that *AO* and *APX* can improve the content of AsA (Zhang et al., 2011a,b).

## Quality Characteristics of *Cucurbitaceae*

The common *Cucurbitaceae* vegetable crops include cucumbers (*Cucumis sativus* L.) and pumpkins (*Cucurbita moschata*), and their morphological characteristics are quite different from each other. Cucumbers with a slender and uniform body are preferred for cooking and fresh eating in China. To make pickled cucumbers, Russians prefer short fruits. Moreover, some people believe that cucumbers with small and dense thorns are better, and those with large and sparse thorns do not have the unique flavor of cucumbers. However, other people like cucumber fruit without thorns and growths. For yellow or green pumpkin, people believe that the darker the pumpkin is, the greater the sweetness. Consumers also find that yellow pumpkin is suitable for steaming or cooking porridge to make various cakes, while green pumpkin is suitable for fried food. In addition to cucumbers and pumpkins, *Cucurbitaceae* vegetables such as melons and watermelons exist in various shapes and colors. Therefore, *Cucurbitaceae* crops of different qualities should be cultivated to meet the demands of consumers.

### Commodity Quality of *Cucurbitaceae*

The main edible part of *Cucurbitaceae* crop species such as cucumber is the fruit. Together with tomato and other *Solanaceae* crops, pericarp color, pulp color, and fruit luster are also important commodity qualities of *Cucurbitaceae* vegetables (Zhang et al., 2019; Gebretsadik et al., 2021; Mashiane et al., 2021). Fruit length and wax powder are the unique commodity qualities of *Cucurbitaceae* crops and have long been the focus of research (Hu et al., 2011; Dou et al., 2018; Ding et al., 2020). For cucumbers, it is difficult to select for fruit firmness during breeding. Peterson et al. found that cucumber firmness was quantitatively inherited with sufficient additive effects, so environmental effects and polygenic heritability were obvious. Fruit length is important to *Cucurbitaceae* crops, especially cucumber. Similar to *Brassicaceae*, some key genes controlling cucumber fruit length have been identified that are related to plant hormones, especially auxin. Knockout of the *FRUITFULL 1* (*CsFUL1*) gene results in further elongation of cucumber fruit, while the high expression of the *CsFUL1* gene significantly shortens cucumber fruit. In addition, *CsFUL1* inhibits the expression of the auxin transporters *PIN-FORMED1* (*PIN1*) and *PIN7*, resulting in a decrease in auxin accumulation in fruit, thereby affecting the fruit length of cucumber (Jiang et al., 2015; Zhao et al., 2019). To clarify the effect of plant hormones on the length of cucumber fruit, researchers can adjust the morphology of high-quality cucumbers in the actual production process. Wax powder is one of the factors affecting the glossiness of fruit. The *AtWAX2* homolog *CsWAX2* in cucumber was cloned and found to be highly expressed in synthetic waxy epidermis. The ectopic expression of *CsWAX2* in the *Arabidopsis wax2* mutant can partially complement the bright stem phenotype (Wang et al., 2015). Additionally, grafting cucumber onto pumpkin rootstock is an effective way to produce glossy cucumber fruits. *AtWIN1* is a regulator of wax biosynthesis that can activate the expression of wax biosynthesis genes such as *CER1*, *CER2*, and *KCS1*; when the expression is enhanced, the mutant *Arabidopsis* became glossier than the wild type (Broun et al., 2004). *CsWIN1* and several key wax biosynthesis genes, including *CsCER1*, *CsCER1-1*, *CsCER*, *4,3-KETOACYL-CoA SYNTHASE* (*CsKCS1*) and the wax transport gene *ATP-BINDING CASSETTE (CsABC)*, are significantly upregulated in cucumber grafted onto pumpkin, so these genes are positively correlated with wax synthesis. More wax esters (C20 fatty acid composition) and fewer alkanes (C29 and C31) were deposited in the grafted cucumber pericarp, probably due to the high wax ester content and the high integration of small trichomes in the pericarp (Zhang et al., 2019). However, there are few studies on the glossiness of *Cucurbitaceae*, and the mechanism needs to be further clarified.

### Flavor Quality of *Cucurbitaceae*

Using cucumber as an example, its flavor quality generally refers to its unique smell and taste (Shimomura et al., 2016). The bitterness of *Cucurbitaceae* is caused by cucurbitacin. The transcription factors *BITTER LEAF* (*BL*) and *BITTER FRUIT* (*BT*) can be used to regulate bitterness formation in leaves and fruits, respectively, by applying the integrative bioinformatics and molecular biology approaches described above. Researchers have identified four additional *P450* genes (*Csa3G698490*, *Csa3G903540*, *Csa3G903550*, and *Csa1G044890*) that are coexpressed with the *BI* cluster (Shang et al., 2014). In addition to bitterness, unique aromatic substances and some non-volatile substances have been reported in cucumber. Researchers used GC–MS to analyze eighty-five volatile chemicals, including thirty-six volatile terpenes in twenty-three different tissues of cucumber, and found that TERPENE SYNTHASE11 (TPS11)/TPS14, TPS01, and TPS15 are responsible for volatile terpenoid production in the roots, flowers, and fruit tissues of cucumber plants and can improve cucumber flavor (Wei et al., 2016). Many kinds of cucumber flavor substances have been identified, of which (E,Z)-2,6-non-adienal (NDE) is an important commercial flavor. NDE has been found in various plant materials, but cucumbers are considered the best source of this flavor substance. NDE production is reduced by acidification, enhanced by linolenic acid, and unaffected by unsaturated fatty acids, NaCl or CaCl_2_ (Buscher and Buscher, 2001). Cucurbitacin is a unique substance of cucumbers. The mechanism of cucumber bitterness must be clarified to combine the non-bitterness characteristics with other excellent characteristics to cultivate high-quality cucumbers.

### Nutritional Quality of *Cucurbitaceae*

Nutritional quality mainly refers to the nutrition and health care ingredients needed by the human body. For cucumber, pumpkin and other *Cucurbitaceae* crops, AsA, soluble solids, soluble protein, soluble sugar and other traits are important for the measurement of nutritional quality (Ge et al., 2017; Gao et al., 2018; de Almeida et al., 2019; Buzigi et al., 2021). ASCORBATE OXIDASE is a copper-containing enzyme localized at the apoplast, where it catalyzes the oxidation of AsA to dehydroascorbic acid (DHA) via a monodehydroascorbic acid (MDHA) intermediate. Similar to solanaceous crops, AO can also affect the content of AsA in *Cucurbitaceae* crops. The reduction in AO activity increases the AsA content in melon (*Cucumis melo* L.) fruit, which is due to the oxidation of AsA and the expression of certain biosynthetic and recycling genes, such as *CmAPX1*, *CmMDHAR*, and *CmDHAR* (Chatzopoulou et al., 2020). Consequently, the ascorbate redox state is altered in the apoplast. Interestingly, transgenic melon, which suppresses AO expression, displays an increased ethylene production rate coinciding with elevated activity and gene expression levels of 1-aminocyclopropane-1-carboxylic acid (ACC) oxidase (ACO), which might contribute to early ripening; moreover, AO participates in the rapid fruit growth of *Cucurbitaceae in vivo* (Chatzopoulou et al., 2020). Sugars provide a strong, pleasant, sweet taste and deliver energy when ingested; appetite regulatory centers respond to the energy content of sugar, which has a major influence on human health (Anderson, 1995). Thus, studies on the soluble sugar content of vegetables are of great significance. Comparing two cultivated pumpkin lines with different sweetness, C. *maxima* inbred lines “98-2” and “312-2,” the glucose content of “312-1” decreased rapidly in the early stage of fruit development, which may have been related to the high expression of sucrose *INVERTASE* (*INV*) and *HEXOKINASE (HK*) at this stage. In contrast, *FRUCTOSE KINASE* (*FK*), which is responsible for fructose metabolism, is differentially expressed at different stages of fruit development. These results suggest that *INV, HK*, and *FK* may serve important roles in promoting sucrose biosynthesis during pumpkin fruit development (Wang et al., 2020).

## Complexity of Vegetable Quality Regulation

**FIGURE 2:**
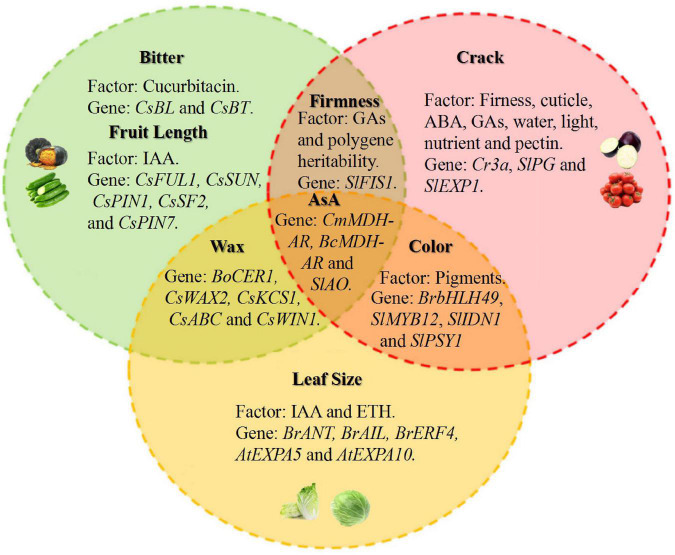
Overview of the factors and genes related to vegetable quality, taking *Brassicaceae*, *Solanaceae* and *Cucurbitaceae* as examples. Phytohormones represent the main factor that can influence fruit length (*Cucurbitaceae*), leaf size (*Brassicaceae*), firmness (*Solanaceae*) and cracking (*Solanaceae*). Cucurbitacin is a unique substance of cucumbers that affects bitterness. Genes regulate different quality characteristics of vegetables, such as fruit length in cucumber (*CsFUL1*, *CsSUN*, *CsPIN1*, *CsPIN7* and *CsSF2*), cracks in tomato (*Cr3a*, *SlPG* and *SlEXP1*) and leaf size in Chinese cabbage (*BrANT*, *BrAIL* and *BrERF4*). Some homologous genes such as *MDHAR* also play similar roles in vegetable quality, which affects the content of AsA in non-heading Chinese cabbage and melon.

From the above, many studies on the genes related to the regulation of vegetable quality have been conducted, and genes that are highly conserved or belong to the same gene family in different varieties are likely to have close functional correlations (Figure 2). For example, MDHAR, the key enzyme in AsA synthesis, can catalyze the reduction of MDHA to AsA (Park et al., 2016). When *MDHAR* was silenced in different species, such as melon, acerola, and Chinese cabbage, the content of AsA decreased (Eltelib et al., 2011). Moreover, *SUN* is one of the main genes controlling tomato fruit shape and encodes a member of the *IQ67-DOMAIN* (*IQD*) family of calmodulin-binding proteins. When *SUN* expressed at high levels in the fruit, tomato showed an elongated shape (Xiao et al., 2008). More precisely, SUN controls tomato shape through redistribution of mass that is mediated by increased cell division in the longitudinal direction and decreased cell division in the transverse direction of the fruit (Wu et al., 2011, 2015). The cucumber *CsSUN* gene is also involved in the regulation of cucumber spherical fruit development, and the round-fruited WI7239 had a 161-bp deletion in the first exon of *CsSUN*. The expression of *CsSUN* in round-fruited WI7239 was significantly lower than that in long-fruited WI7238 (Pan et al., 2017). Although *SUN* has some effect on the fruit shape of tomato and cucumber, *SUN* affects the length of tomato and changes the width of cucumber fruits. It is necessary to understand the specific mechanism of *SUN* in different crops. Thus, although the genes regulating quality traits are conserved in different vegetables, their mechanisms of action remain to be further confirmed.

Constituting a common factor in vegetable quality regulation, phytohormones have diverse effects on vegetable quality. The same hormones can regulate the same phenotypes in different species, but the mechanisms are not consistent; for example, auxin can influence vegetable leaf or fruit size. *CsFUL1* inhibited the expression of the auxin transporters *PIN1* and *PIN7*, resulting in a decrease in auxin accumulation in fruit, thereby affecting the fruit length of cucumber (Zhao et al., 2019). *BrANT* responds to auxin and regulates downstream gene expression of organogenesis and cell proliferation, indicating that ANT promotes leaf growth by regulating cell division (Ding et al., 2018). These observations suggest that auxin controls many aspects of vegetable organ development in different ways (Pattison et al., 2014). Additionally, the same quality traits of vegetables may be affected by different phytohormones. Taking tomato firmness as an example, Moneymaker (MM) tomato has a higher compression resistance than *Solanum lycopersicum* var. *cerasiforme* LA1310 (CC). Compared with the fruits of the near-isogenic line with the CC *qFIRM SKIN 1* (q*FIS1*) allele (NIL-*FIS1^CC^*) Li *et al.* found that bioactive GAs, including GA1, GA3, and GA7, were dramatically increased, whereas the metabolic products of GA2-oxidases, including GA8 and GA34, were decreased in the fruits of the near-isogenic line with the MM q*FIS1* allele (NIL-*FIS1^MM^*) (Li et al., 2020). By spraying exogenous GA3 on NIL-*FIS1^CC^* mature green fruits, researchers found significantly increased compression resistance in treated fruits compared to untreated controls, indicating that GA levels contribute to higher fruit firmness (Li et al., 2020). Moreover, researchers treated wild type tomato fruit with exogenous ABA and found that fruit firmness decreased, and the suppression of *9-CIS-EPOXYCAROTENOID DIOXYGENASE* (*SlNCED1*), which encodes a key enzyme in ABA biosynthesis, significantly induced tomato fruit firmness (Sun et al., 2012). GA and ABA can both affect tomato firmness, but the specific mechanism of action and the connection between the two require further analysis. Furthermore, there is extensive cross-talk between different phytohormones that control growth and development, such as auxin and GA. AUXIN RESPONSE FACTORS (ARFs) are transcription factors that respond to auxin signals and activate or repress downstream gene expression, thus delivering the signal for the regulation of a set of genes (Roosjen et al., 2018). As critical transcriptional downstream targets of *ARF2*, *CARBON-METABOLISM INVOLVED* (*GNC*) and *GNC-LIKE* (*GNL*) are involved in plant greening, flowering time, and senescence (Richter et al., 2013). It has been reported that the constitutive activation of GA signaling is sufficient to suppress *arf2* mutant phenotypes, causing increased chlorophyll accumulation and delayed senescence through repression of *GNC* and *GNL* (Richter et al., 2013). These and other aspects of different pathways suggest complex crosstalk in multiple phytohormone signals, reflecting their pleiotropic effects on vegetable growth and ripening. Finally, phytohormones act as common factors regulating vegetable quality, and deepening the mechanistic understanding of molecular events related to phytohormones will help to elucidate the regulatory networks that affect vegetable quality.

## Genetic Manipulation Applied in Vegetable Quality

Many researchers have identified the key genes that affect vegetable qualities. For example, Wiesner et al. (2014) identified genes coding for polypeptide four of the cytochrome P450 (CYP) monooxygenase subfamily CYP81F and examined their metabolic roles in indole glucosinolate biosynthesis in *Brassica rapa* ssp. *chinensis* by microarray. Li et al. (2016) found that exogenous auxin alters the expression patterns of ethylene and auxin signaling-related genes that are induced or repressed in the normal ripening process by Illumina RNA sequencing. Kompetitive allele-specific PCR (KASP) is a proprietary technology of LGC genomics that can distinguish alleles at variant loci (Semagn et al., 2014; Steele et al., 2018). Paudel et al. (2019) used the four loci to facilitate marker-assisted selection (MAS) for watermelon seed coat color. The results of these studies can be applied to actual production processes to improve vegetable quality. With the development of molecular biology technology, the regulation of vegetable quality is not confined to traditional methods, such as grafting techniques and the application of exogenous fertilizers, but regulation at the molecular level is becoming increasingly common.

DNA molecular marker technologies are marker techniques based on nucleotide differences between individuals. This refers to genetic markers based on DNA. In other words, direct response to genetic material variation at the DNA level may accurately reveal interspecific and intraspecific differences (Vieira et al., 2007; Alim et al., 2011; Ferguson et al., 2012; Xu et al., 2019; El-Mansy et al., 2021).

DNA molecular markers are widely used in vegetable breeding research, including the construction of genetic maps to facilitate the selection of breeding parents, the stable and objective analysis of genetic relationships and plant origin and evolution, and the mapping of agronomic trait genes (Table 1). Restriction fragment length polymorphism (RFLP) is the earliest DNA molecular marker technology. By mapping chromosome-specific tomato RFLP markers in potato and conversely mapping potato markers in tomato, the different potato and tomato RFLP maps were aligned, and the similarity of the potato and tomato genomes was confirmed (Gebhardt et al., 1991). RFLP has also been used for the identification of S-haplotypes of breeding lines in broccoli and cabbage (*B. oleracea* L.) and in purity tests of F_1_ hybrid seeds (Sakamoto et al., 2000). Simple sequence repeat (SSR) markers have been widely used to obtain new germplasm in recent years. When 32 SSR pairs of primers derived from the *A. thaliana* chloroplast genome and another 21 SSR primers from the *B. napus* mitochondrial genome sequences were compared, six types of cabbage cytoplasmic male sterility (CMS) were found, namely, NigCMS, OguCMSR (1), OguCMSR (2), OguCMSR (3), OguCMSHY, and PolCMS (Wang et al., 2012). Additionally, Gharsallah et al. (2016) genotyped cultivars using nineteen polymorphic SSRs out of twenty-five tested to produce a total of seventy alleles with an average of 3.68 alleles per locus and polymorphism information content (PIC) values ranging from 0.22 to 0.82 in saline tolerant, mildly tolerant and saline sensitive tomatoes, so SSR marker-genotypes can be used to find potential salt tolerance sources in tomato. Furthermore, the single nucleotide polymorphism (SNP) markers screened in tomato cultivar lines can be used to estimate the transferability of these SNPs to other breeding materials (Shirasawa et al., 2010). A core set of twenty-four SNPs can distinguish 99% of the two hundred and sixty-one cucumber varieties (Zhang et al., 2020a). These methods can be used comprehensively. For example, amplified fragment length polymorphism (AFLP) and SSR techniques combined with bulk segregant analysis (BSA) can be used to map the *RESTORER GENE* (*BrRFP*) in heading Chinese cabbage using the F_2_ segregating population developed by crossing the polima (pol)-like CMS line 06J45 and the restorer line 01S325 (Xu et al., 2014). Ohyama et al. (2017) identified thirteen important agronomic quantitative trait loci (QTLs) in tomato by SSR molecular markers. Xu et al. (2019) developed thirty-five informative InDel markers that were successfully used to analyze the genetic diversity of thirty-six cabbage germplasms, providing molecular marker data for genetic mapping and germplasm identification and promoting genetic improvement in cabbage breeding.

**TABLE 1:** Different types of molecular markers.

Type	Molecular markers	References
Molecular hybridization	RFLP	Gebhardt et al., 1991; Sakamoto et al., 2000
PCR	SSR SNP	Shirasawa et al., 2010; Wang et al., 2012; Gharsallah et al., 2016; Ohyama et al., 2017; Paudel et al., 2019; Zhang et al., 2020a
PCR and Restriction Enzyme Digestion Technology	AFLP	Xu et al., 2014

### Transgenic Technology to Regulate the Quality of Vegetables

Transgenic technology involves the use of modern biotechnology to artificially separate, recombine, introduce, and integrate required target genes into the genome of organisms, thereby improving the original traits or providing new desirable traits (Kumar et al., 2020). Given that the essence of transgenic technology and traditional technology is genetic improvement through the acquisition of excellent genes, the close combination of transgenic technology and conventional breeding techniques can enable breeding of new varieties with multiresistance, high quality, high yield and high efficiency. This approach can greatly improve the efficiency of variety development, reduce the input of pesticides and fertilizers, and enable great potential in alleviating resource constraints, ensuring food safety, protecting the ecological environment and expanding agricultural functions (Ndimba et al., 2017).

Transgenic experiments play an important role in the verification of gene function (Table 2). The tomato genes *ATP-BINDING CASSETTE TRANSPORTER G 36* (*SlABCG36*) and *SlABCG42*, which encode proteins that are highly homologous to *AtABCG32*, can be downregulated using RNAi techniques, resulting in the deposition of the stratum corneum in the tomato fruit and the formation of a thinner stratum corneum; ABCG transporter protein affects the transport of keratin precursors (Elejalde-Palmett et al., 2021). Zhang et al. (2020b) used *A. thaliana* as a material and overexpressed *AtMYB49* to improve the ability of plants to resist oxidative stress. Arce-Rodriguez and Ochoa-Alejo designed a virus-induced gene silencing (VIGS) system for *Capsicum*, providing a new means for the study of gene function in this genus (Arce-Rodriguez and Ochoa-Alejo, 2020). The pepper color genes *CAPSANTHIN/CAPSORUBIN SYNTHASE* (*CCS*), PHYTOENE SYNTHASE (PSY), *LYCOPENE-BETA-CYCLASE* (*LCYB*), and *BETA-CAROTENE HYDROXYLASE* (*CRTZ*) were silenced separately through VIGS technology, and pepper fruits from red fruit cultivars turned orange or yellow. Moreover, these four genes were silenced simultaneously; the fruits did not show the normal red color ation (Tian et al., 2014). Recently, researchers applied VIGS of pepper genes via a pTRV2-GFP-CaPDS vector, which can visualize TRV spread and monitor VIGS efficiency, thereby enriching the research methods for vegetable germplasm resources (Zhou et al., 2021).

**TABLE 2:** Application of transgenic and genome editing technology in vegetables.

Type	Method	Gene	Quality	Challenges
Transgenic technology	RNAi	*SlABCG36 SlABCG42*	Tomato has a thinner stratum corneum	The safety of genetically modified vegetables is still unclear
	VIGS	*CaCCS CaPSY CaLCYB CaCRTZ*	Pepper fruits turn orange or yellow	
Genome editing technology	CRISPR	*SlFIS1*	Increase tomato firmness	Traits do not coexist with others
		*CsSF2*	Shoot cucumber fruit	The serious growth inhibition of homozygous plants

### Gene Editing Technology to Regulate the Quality of Vegetables

Gene editing, also known as genome editing or genome engineering, is a relatively accurate genetic engineering technology or process that can modify specific target genes in the genome of organisms (Schindele and Puchta, 2020). Clustered regularly interspaced short palindromic repeats/Cas (CRISPR/Cas) technology is the third generation of genome editing technology developed in recent years. It is a means to study gene function and improve crop yield. Gene editing shows great potential in gene research and genetic improvement because it can efficiently edit the targeted genome (Li et al., 2021).

At present, CRISPR technology has been applied to the targeted editing of genomes of various organisms (Table 2). Li et al. (2020) used CRISPR/Cas9 technology to edit *FIS1* to verify its effect on tomato firmness (Li et al., 2020). *SHORT-FRUIT 2* (*SF2*) encodes histone deacetylase proteins, which participate in diverse and tissue-specific developmental processes by forming various corepressor complexes with different regulatory subunits. Researchers knocked out the *SF2* gene by CRISPR/Cas9 and proved that *SF2* controls fruit cell proliferation by targeting the biosynthesis and metabolism of cytokinin and polyamines (Zhang et al., 2020c).

## Perspectives

Currently, there are new demands for vegetable quality in terms of diversity and nutritional health. Improving vegetable quality is an important topic in contemporary vegetable research. Scientists have conducted in-depth research on the molecular development, regulatory mechanisms and biosynthesis of vegetable quality traits. Studies have mainly included the identification of candidate genes, and their loci, which are responsible for fruit quality traits of different varieties of vegetables, as well as the physiological and metabolic pathways that directly or indirectly affect fruit quality traits. In addition, the establishment of efficient genetic transformation methods and the use of gene-editing technology help verify the function of these determinants or regulators. These applications for improving quality attributes make sense when traditional breeding is difficult because of the reproductive isolation between species.

Studies have shown that molecular breeding combined with genome editing technology is growing rapidly, and that this approach can shorten the breeding cycle and greatly improve breeding efficiency, which has become a new direction in vegetable breeding (Ueta et al., 2017). Genome editing has been generally applied in horticulture, and gene edited crops mainly include *Solanum lycopersicum, Solanum pimpinellifolium, Solanum tuberosum, Brassica oleracea, Brassica napus, Brassica carinata, Lettuce sativa, Cucumis sativus, Camelina sativa, Daucus carota*, etc. (Xu et al., 2019). Through the application of genome editing technology, new germplasms have been created, such as lettuce with high AsA and strawberry with different sugar contents, and *de novo* domestication of tomato was realized (Li et al., 2018; Zhang et al., 2018; Xing et al., 2020). However, gene manipulation technologies still face challenges in improving vegetable quality. However, whether or not gene-edited vegetables should be regulated as transgenic vegetables has aroused international controversy (Hashimoto et al., 1999; Friedrichs et al., 2019). Thus, gene-edited vegetables are in urgent need of policy support to promote the industrialization of new technologies and products. On the other hand, the precise regulatory mechanisms of complex vegetable traits still need to be further explored. Important agronomic traits might be regulated by multiple gene loci, and one gene may be involved in multiple agronomic traits. For example, *SF2*, which encodes histone deacetylase protein, not only affects fruit quality but also participates in the normal growth of plants. The cucumber *sf2* knockout mutant exhibits severely hindered growth and cannot develop further. The application of CRISPR technology can only be used to analyze the functional mechanism by which *CsSF2* regulates cucumber fruit length and cannot be applied to the actual production process (Zhang et al., 2020c). Therefore, one of the biggest challenges facing future gene manipulation technologies is to identify precise trait regulatory networks and disrupt the unwanted linkages between different traits.

Furthermore, the development of genomics, molecular biology, imaging, remote sensing informatics, and big data technology will promote the development of breeding science (Mahlein et al., 2012; Boukar et al., 2019; Walter et al., 2019; Zhao et al., 2019). In particular, the application of big data technology in gene breeding plays a huge role in screening for functional variants, since it improves the efficiency and accuracy of variant detection. The target of genetic variation detection in plants has shifted from single SNP to structural variation and insertion or deletion allelic variation though big data technology (Wong et al., 2019). In summary, although challenges remain, the application of genetic manipulation in horticultural crop species improvement will further create and enhance vegetable quality through the inclusion of desirable traits.

## Author Contributions

LG and NH analyzed the gene manipulation effects on the quality of vegetables. TW and JC conceived the original idea for the review. All authors wrote the manuscript.

## Conflict of Interest

The authors declare that the research was conducted in the absence of any commercial or financial relationships that could be construed as a potential conflict of interest.

## Publisher’s Note

All claims expressed in this article are solely those of the authors and do not necessarily represent those of their affiliated organizations, or those of the publisher, the editors and the reviewers. Any product that may be evaluated in this article, or claim that may be made by its manufacturer, is not guaranteed or endorsed by the publisher.
